# *Bacillus anthracis *secretome time course under host-simulated conditions and identification of immunogenic proteins

**DOI:** 10.1186/1477-5956-5-11

**Published:** 2007-07-27

**Authors:** Alexander Walz, Cesar V Mujer, Joseph P Connolly, Tim Alefantis, Ryan Chafin, Clarissa Dake, Jessica Whittington, Srikanta P Kumar, Akbar S Khan, Vito G DelVecchio

**Affiliations:** 1 Vital Probes, Inc., 1300 Old Plank Road, Mayfield, PA 18433, USA; 2 BAE Systems Inc., 164 Totowa Road, Wayne, NJ 07474, USA; 3 Chemical and Biological Defense Directorate, Defense Threat Reduction Agency, 6801 Telegraph Road, Alexandria, VA, USA; 4 Calvert Laboratories, Inc., Scott Technology Park, 100 Discovery Drive, Olyphant, PA 18447, USA

## Abstract

**Background:**

The secretion time course of *Bacillus anthracis *strain RA3R (pXO1^+^/pXO2^-^) during early, mid, and late log phase were investigated under conditions that simulate those encountered in the host. All of the identified proteins were analyzed by different software algorithms to characterize their predicted mode of secretion and cellular localization. In addition, immunogenic proteins were identified using sera from humans with cutaneous anthrax.

**Results:**

A total of 275 extracellular proteins were identified by a combination of LC MS/MS and MALDI-TOF MS. All of the identified proteins were analyzed by SignalP, SecretomeP, PSORT, LipoP, TMHMM, and PROSITE to characterize their predicted mode of secretion, cellular localization, and protein domains. Fifty-three proteins were predicted by SignalP to harbor the cleavable N-terminal signal peptides and were therefore secreted via the classical Sec pathway. Twenty-three proteins were predicted by SecretomeP for secretion by the alternative Sec pathway characterized by the lack of typical export signal. In contrast to SignalP and SecretomeP predictions, PSORT predicted 171 extracellular proteins, 7 cell wall-associated proteins, and 6 cytoplasmic proteins. Moreover, 51 proteins were predicted by LipoP to contain putative Sec signal peptides (38 have SpI sites), lipoprotein signal peptides (13 have SpII sites), and N-terminal membrane helices (9 have transmembrane helices). The TMHMM algorithm predicted 25 membrane-associated proteins with one to ten transmembrane helices.

Immunogenic proteins were also identified using sera from patients who have recovered from anthrax. The charge variants (83 and 63 kDa) of protective antigen (PA) were the most immunodominant secreted antigens, followed by charge variants of enolase and transketolase.

**Conclusion:**

This is the first description of the time course of protein secretion for the pathogen *Bacillus anthracis*. Time course studies of protein secretion and accumulation may be relevant in elucidation of the progression of pathogenicity, identification of therapeutics and diagnostic markers, and vaccine development. This study also adds to the continuously growing list of identified *Bacillus anthracis *secretome proteins.

## Background

*Bacillus anthracis *is a Gram-positive spore-forming bacterium that is the etiologic agent of anthrax [1]. During the course of infection, the virulent spores germinate to become vegetative cells. The bacterium secretes two major virulence factors, the bipartite edema and lethal toxins that are encoded by plasmid pXO1 [1-3]. The common component of both toxins is the protective antigen (PA) which is not toxic by itself. PA binds a specific receptor on the host cells and translocates the edema factor (EF) and lethal factor (LF) inside the cells where they exert their damaging action. Transcription of the genes coding for these virulence factors has been shown to be coordinately induced by bicarbonate-CO_2_. High CO_2 _tension is believed to simulate conditions encountered within the host [4]. In addition, the effect of temperature has been shown to be important for toxin production but not for production of antiphagocytic capsule, whose synthesis is encoded by genes on plasmid pXO2 [5]. Thus, when *B. anthracis *is cultured at 37°C in a bicarbonate-containing minimal medium, toxin production is enhanced.

The composition and identification of *B. anthracis *extracellular proteins (secretome) has been the subject of recent proteomic studies [6-9]. In *B. cereus*, a close relative of *B. anthracis*, most of these secreted proteins include collagenases, phospholipases, hemolysins, proteases, and enterotoxins that are positively regulated by *plcR *[10]. However, a non-sense mutation inactivates a homolog of this gene in *B. anthracis *resulting in a significant reduction of these secreted proteins [1,11,12]. Using 2-DE and MALDI-TOF MS, Chitlaru *et al*. [6] have identified 64 extracellular proteins in a virulent *B. anthracis *strain, of which 50 exhibit export signal peptides. Thirty-one of these secreted proteins harbor features that are characteristic of virulence determinants suggesting that in addition to the "classic" lethal and edema toxins, a large number of proteins may be essential for *B. anthracis *virulence [6]. Additionally, in minimal medium under high CO_2 _tension, the presence of the plasmids led to the enhanced secretion of 12 chromosome-encoded and 5 pXO1 encoded proteins. Ten of the chromosome-encoded proteins could not be detected in the absence of the plasmids. These results suggest distinct plasmid and chromosome CO_2_-dependent crosstalk mechanisms that modulate extracellular proteolytic activities. Likewise, Antelmann *et al*. [7] have identified 64 extracellular proteins in the non-virulent *B. anthracis *strain UM23C1-2 (pXO1^-^/pXO2^-^), 29 of which were predicted to be secreted. The remainder of the extracellular proteins were predicted to be associated with the cell wall or the cytosol. Based on the nature of *B. anthracis *secretome, it was suggested that this organism is adapted to life in a protein-rich environment due to the presence of a variety of proteases, peptidases, peptide-binding proteins, as well as enzymes required for the metabolism of amino acids. It was also proposed that these secreted proteases and peptidases could be useful targets for the development of improved vaccines.

In another study, Gohar *et al*. [8] compared the fully cured *B. anthracis *extracellular proteomes to two other members of the *B. cereus *group, *B. cereus *and *B. thuringiensis*, that were also cured of their plasmids. The secretomes of the three species included both cell wall and cytosolic proteins that are similar in all three species. However, whereas the extracellular proteins of *B. cereus *and *B. thuringiensis *contained a large number of secreted proteins such as degradative enzymes and toxins, those of *B. anthracis *contained only one secreted protein, a metalloprotease InhA1. These results are in contrast to those reported by Chitlaru *et al*. [6] and Antelmann *et al*. [7] where 31 and 29 secreted proteins were identified in the culture filtrate of fully cured *B. anthracis *cells, respectively. Using minimal medium under high CO_2 _tension, a comparative analysis of the extracellular proteomes of three isogenic strains of *B. anthracis *that differed solely in their plasmid content was conducted by Lamonica *et al*. [9]. In this study, the use of SignalP for the prediction of cellular function and location of each protein indicated that most of the identified proteins were either cell wall-associated or cytoplasmic in the fully cured strain, RA3:00.

The present study characterized the time course of protein secretion and accumulation in the culture of *B. anthracis *strain RA3R (pXO1^+^/pXO2^-^). Secretion patterns at the different time points are due not only to differentially expressed proteins at various phases of exponential growth, but also to the accumulation of proteins in the culture and their stability therein. Particular focus was placed on the analysis of protein secretion during the exponential growth phase to preclude the identification of proteins that may have been caused by bacterial lysis. In addition, computer assisted comparative analysis using 2D Phoretix of the cytosol with the secretome at16 hr under induced conditions was performed. LC MS/MS was used as a complementary tool in identifying proteins that were not amenable to MALDI-TOF MS, significantly increasing the number of identified secreted proteins. The data from this analysis add to the growing list of identified proteins in the secretome database of *B. anthracis *and help in further identifying key pathways associated with virulence. Furthermore, immunogenic proteins, or the immunome, of the secretome were identified using sera from human patients infected with cutaneous anthrax. Identification of these immunodominant antigens is essential for development of more efficacious and safer vaccines against anthrax.

## Results

### Time course of protein secretion

**Figure 1:**
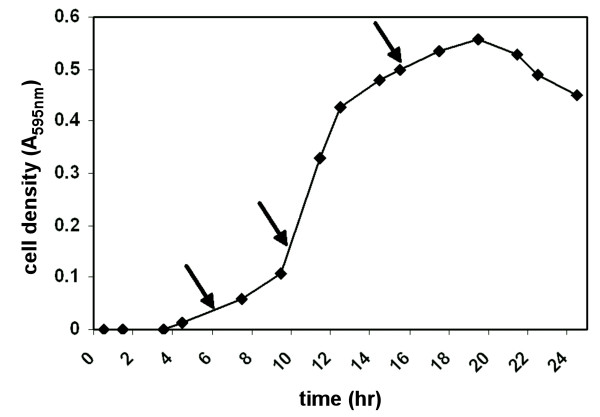
Growth curve for *B. anthracis *strain RA3R (pXO1^+^/pXO2^-^) in R medium under host simulated conditions (induced). Secretome proteins were harvested at time points 6, 10, and 16 hrs as indicated by the arrows.

**Figure 2:**
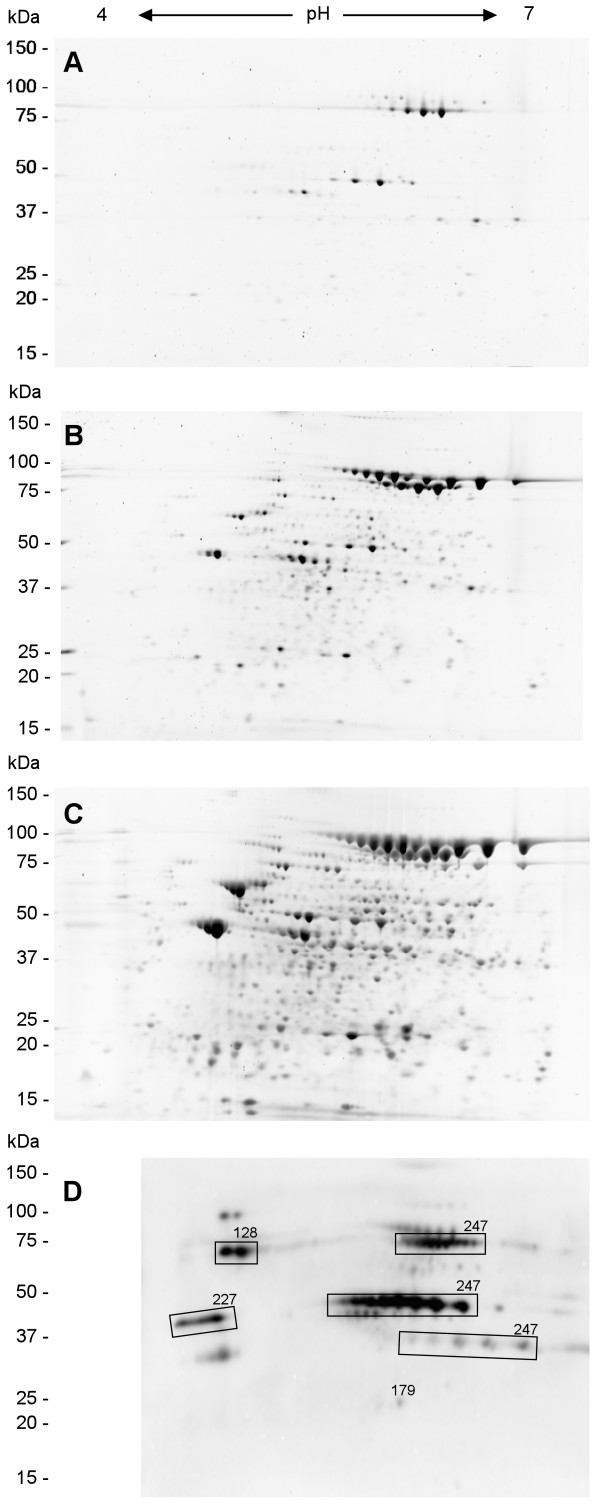
2-DE gel images of SYPRO Ruby-stained secretome proteins of toxigenic, non-encapsulated *B. anthracis *strain RA3R (pXO1^+^/pXO2^-^) from pH 4 to 7 at 6 hr (A), 10 hr (B) and 16 hr (C) after inoculation under conditions that simulate those found inside the host (induced), and (D) Western blot analysis of immunogenic extracellular proteins at 16 hr using sera from patients infected with cutaneous anthrax. Spot numbers refer to additional file 1.

**Figure 3:**
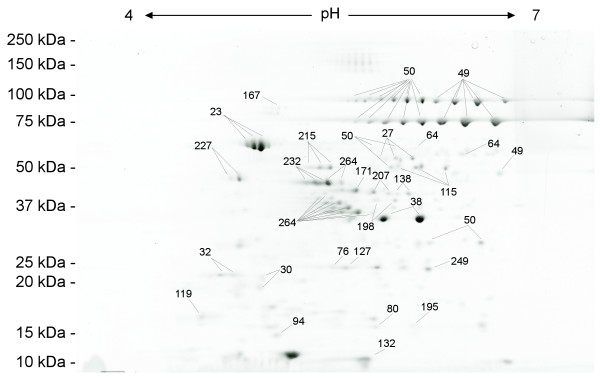
Annotated 2-DE gel image of SYPRO Ruby-stained secretome proteins of *B. anthracis *strain RA3R (pXO1^+^/pXO2^-^) at 16 hr after inoculation under laboratory conditions (uninduced). Spot numbers refer to additional file 1.

**Figure 4:**
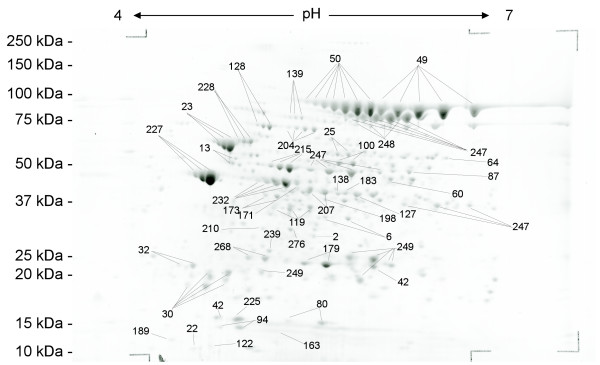
Annotated 2-DE gel image of SYPRO Ruby-stained secretome proteins of *B. anthracis *strain RA3R (pXO1^+^/pXO2^-^) at 16 hr after inoculation under host simulated conditions (induced). Spot numbers refer to additional file 1.

**Figure 5:**
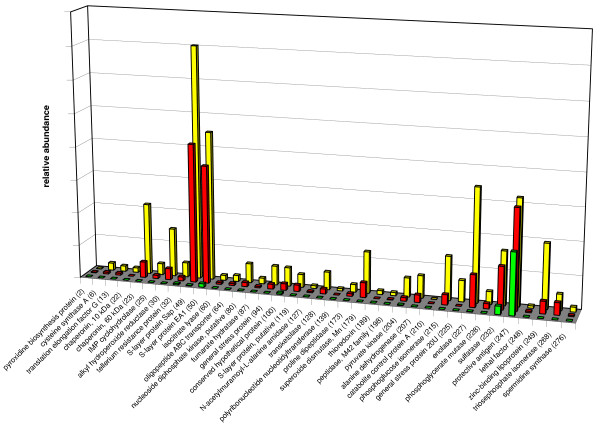
Relative protein expression of differentially expressed extracellular proteins of *B. anthracis *strain RA3R (pXO1^+^/pXO2^-^) during exponential growth at 6 hr (green bars), 10 hr (red bars) and 16 hr (yellow bars), based on spot size and pixel intensity. The relative amount of each protein was determined using the 2D Phoretix software. The protein numbers refer to additional file 1.

The extracellular proteomes of *B. anthracis *strain RA3R were analyzed at three time points during the exponential growth phase, *i.e.*, early (6 hr), mid (10 hr) and late log (16 hr) under induced, host-simulated conditions (Fig. 1). 2D Phoretix computer-assisted analysis of 2-DE average gels revealed that the number of protein spots increased from 54 spots at 6 hr to 308 spots at 10 hr and to 536 spots at 16 hr. (Figs. 2A–C). In contrast, the number of protein spots at 16 hr under uninduced condition (Fig. 3) is only 178 or 33% of the total number under induced condition (Fig. 4). The S-layer proteins Sap (surface array protein) and EA1 (extractable antigen 1), PA, and enolase were the predominant proteins that increased in secretion (Fig. 5). Other highly expressed proteins include alkyl hydroperoxide reductase, chaperonin 60kDa, phosphoglycerate isomerase, sulfatase, manganese superoxide dismutase, and a zinc-binding lipoprotein.

### Extracellular proteins

A total of 275 proteins were identified in the extracellular proteome by a combination of MALDI-TOF MS and LC MS/MS (Fig. 3/4, additional file 1). MALDI-TOF MS analysis has identified 52 proteins of which 47 were also identified by LC MS/MS analysis. In contrast, 270 proteins were identified by LC MS/MS. This indicates that the combined use of MALDI-TOF MS and LC MS/MS has significantly increased the total number of identified proteins in the extracellular proteome, which has also been observed in the analysis of the proteome of other bacteria such as *Mycobacterium tuberculosis *[13] and *Brucella abortus *[14].

**Figure 6:**
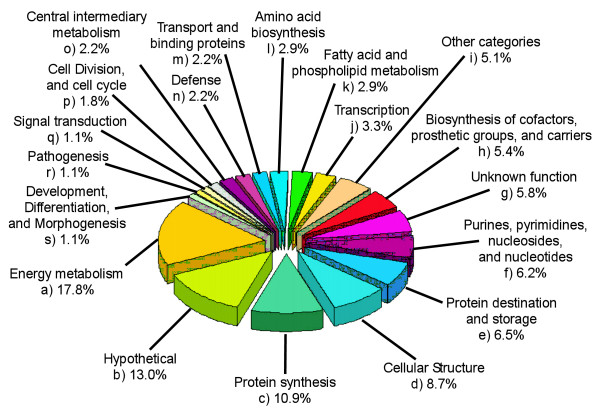
Role category pie chart showing the percentage of all identified *B. anthracis *strain RA3R (pXO1^+^/pXO2^-^) secretome proteins in each role family.

The *B. anthracis *secretome proteins were also grouped into major cellular functions (Fig. 6). Half of all identified secretome proteins are involved in energy metabolism (17.8%), protein synthesis (10.9%), cellular structure (8.7%), or are hypothetical (13%). This distribution is significantly different from role category predictions of all genes theoretically encoded by *B. anthracis*. Some groups were overrepresented or underrepresented relative to the theoretical total *B. anthracis *proteome, where energy metabolism accounts for 5.2%, protein synthesis is 2.4%, cellular structure is 7.1%, and hypothetical proteins are 40.6% of all cellular proteins. The role category predictions of *B. anthracis *were obtained from The Institute for Genomic Research website [15].

**Figure 7:**
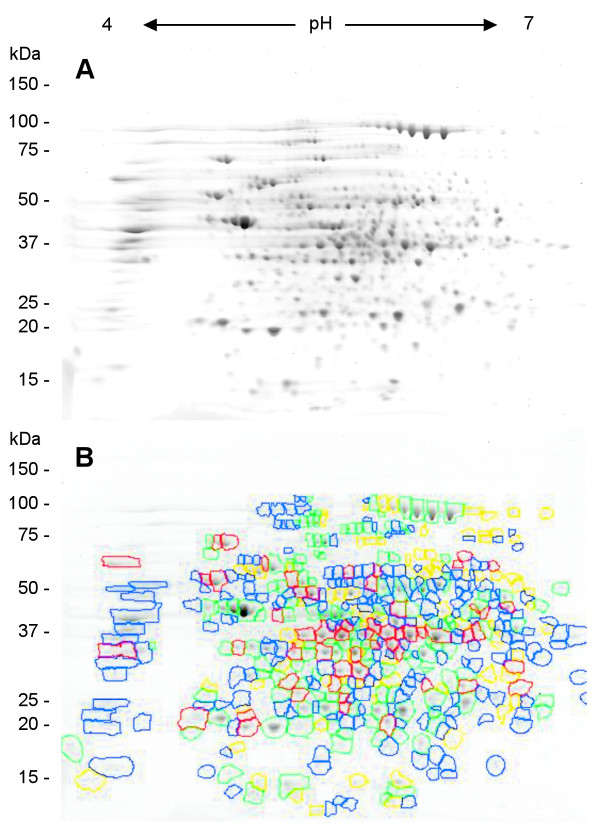
(A) 2-DE gel image of SYPRO Ruby-stained cytosol proteins of toxigenic, non-encapsulated *B. anthracis *strain RA3R (pXO1^+^/pXO2^-^) from pH 4 to 7 at 16 hr under induced conditions. (B) 2D Phoretix comparative analysis of the cytosol with the secretome at 16 hr under induced conditions. Proteins encircled in red are more than two fold up-regulated, proteins encircled in yellow are more than two fold down-regulated, proteins encircled in green are between these limits, and proteins encircled in blue are unmatched between the two sub proteomes.

Some of the secretome proteins have metabolic functions that would typically place them in the cytoplasm. However, 2-DE secretome and cytosol subproteomes show distinct, significantly different patterns from another (Fig. 7). Using MALDI-TOF MS the protein spots in the cytosol were identified, the relative protein spot intensities analyzed, and their ratio to each other determined. These results were used for computer assisted comparison of cytosol and secretome 2-DE gels. Out of the 489 unique protein spots in the cytosol, 56 are more than two-fold up regulated, 84 are more than two-fold down regulated, and 177 proteins spots have no corresponding spot in the secretome (Fig. 7B). If protein spots in the secretome were simply the result of cell lysis, not only should their relative position on the gel be the same between the cytosol and the secretome, but the ratio of the proteins to each other in each subproteome should be the same in both. The results show that two thirds of all protein spot positions as well as relative abundances are distinctively different between the cytosol and secretome. Therefore, cell lysis can be excluded as the major contributor to the protein accumulation in the secretome. The extracellular proteins were further analyzed using various bioinformatics software programs, such as SignalP, SecretomeP, PSORT, LipoP, TMHMM, and PROSITE for predicting protein secretion and localization.

### Classical Sec pathway

A total of 53 proteins were predicted by SignalP to be secreted in the classical Sec pathway, which is characterized by the presence of a signal peptide [16,17] (additional file 1). Of the 53 proteins containing the signal peptides, 38 proteins have the cleavage site for signal peptidase I (SpI). These proteins are predicted to be secreted into the external environment, because they lack additional retention signals. However, 10 proteins have the cleavage site for signal peptidase II (SpII) and the retention signals for lipid anchors while three proteins were predicted to have transmembrane helices (TMH). These proteins are predicted to have an extracytoplasmic but cell-associated location. LipoP predictions discriminate between lipoprotein signal peptides, other signal peptides, and N-terminal membrane helices with 93% accuracy in Gram positive bacteria [18]. In contrast to LipoP, TMHMM predicted 25 integral membrane proteins with one to ten transmembrane helices [19] (additional file 1). TMHMM predicts transmembrane protein topology with a hidden Markov model with a 98% accuracy. Additional predictions using PROSITE identified several secreted proteins that are also know to be associated with the bacterial cell wall by S-layer homology domains or lipoprotein lipid attachment sites [7,20]. No cell wall associated proteins with LPXTG-motifs were found in the secretome. Despite its name, the LPXTG-motif cell wall anchor domain protein does not contain such a domain. In fact, it contains a NEAT domain that might be involved in the transport of iron.

### Alternative Sec pathway

Twenty-three proteins were predicted by SecretomeP to be secreted by the non-classical Sec Pathway characterized by the lack of typical export signals [21]. In contrast to the predictions of SignalP and SecretomeP, PSORT [22] predicted 171 extracellular proteins, 7 cell wall-associated proteins, and 6 cytoplasmic proteins. Using the whole genome of *B. anthracis*, Binnewies *et al*. have calculated that the *Bacillus anthracis *Ames strain has 6% secreted proteins predicted using SecretomeP, 3% using LipoP, and 6% using SignalP [23].

### Immunogenic extracellular proteins

The main immunogenic proteins detected by 2-DE Western blot analysis using sera from humans infected with cutaneous anthrax include the 83 and 63 kDa charge variants of protective antigen (PA), followed by charge variants of enolase and transketolase (Fig. 2D). The protective antigen produced *in vivo *has a molecular mass of 83 kDa and is subsequently cleaved by cell-associated protease activity resulting in a 63 kDa protein that binds the lethal factor to form lethal toxin [24]. Notably, 17 charge and mass variants of PA were detected. These include five charge variants of the 83 kDa PA isoforms, seven charge variants of the 63 kDa PA isoforms and five charge variants of the 37 kDa PA isoforms. Charge variants of PA have been reported previously. Their generation was due to the spontaneous deamidation of asparagine residues which is dependent on pH, temperature, primary sequence ("nearest neighbor" effect) and protein conformation [25]. Charge variants of enolase and transketolase were also noted. Enolase has also been reported previously as component of the *B. anthracis *spore [26] and as one of the immunodominant spore antigens [27]. Other minor immunogenic proteins were also detected by 2-DE Western blot analysis but were not identified.

## Discussion

The secretome of *B. anthracis *has been the subject of recent proteomic studies [6-9]. Interest in its study comes from the fact that several secreted proteins of *B. anthracis *are known virulence factors (*e.g.*, PA, EF, LF). Other secreted proteins may potentially be involved in the adherence of the bacteria to host cells while some may be required for the suppression of the host's defense mechanisms. While several of the virulence proteins have been identified by recent proteomic studies [6,7], the time course of protein secretion by a toxigenic but non-encapsulated strain RA3R (pXO1^+^/pXO2^-^) under host simulated conditions has not been reported. In this study, the dynamics of secretion of several proteins were investigated at different time points during the exponential growth phase of *B. anthracis*. The same collection points have been used previously by Lamonica *et al*. [9] for the isolation of secretome proteins. Chitlaru *et al*. [6] and Antelmann *et al*. [7] isolated secretome proteins at analogous growth stages as well. As shown in Fig. 5, the rate of protein secretion varies for each protein. It should be noted that at 6 hours, PA has the highest rate of secretion of all secreted proteins. Since PA is a major component of the anthrax toxin, its high secretion rate at this early time point confirms the critical role this protein plays during the onset of anthrax pathogenesis. Two other proteins that were detected in high abundance in the culture supernatant are the S-layer proteins, EA1 and Sap, both of which are components of the cell wall. Both proteins contain a signal peptide followed by three SLH (S-layer homology) anchoring domains and are considered major surface antigens. The amount of these two proteins was highest at the 16-hour time point compared to the other identified extracellular proteins. It has been previously described that Sap is sequentially replaced by EA1 [28]. This rapid S-layer turnover results in the release and spillover of these proteins into the secretome.

Several of the secretome proteins have metabolic functions that would typically place them in the cytoplasm. This is similar to the results by Antelmann *et al*. [7] in which more than half of the identified secretome proteins were associated with the cell wall or cytosol. We also identified 25 proteins that were predicted to have transmembrane helices which would typically associate them to the cell wall. All but one of these proteins were predicted to be secreted by SignalP or SecretomeP and half of these proteins have also been identified before as a natural component of the secretome (see additional file 1).

### Extracellular accumulation of *B. anthracis *proteins

Combined analysis using SignalP and SecretomeP of the *B. anthracis *secreted proteins indicated that 28% of the detected proteins are extracellular whereas 62% are predicted to be extracellular using PSORT. The remaining proteins were predicted to be cytoplasmic since they lacked known export signals or are cell-associated because they have membrane-anchoring or cell wall retention signals. Using LipoP, some of the proteins are predicted to be bound at the *trans *surface of the cytoplasmic membrane. Contrary to their predicted location, a number of proteins with retention signals for covalent or non-covalent attachment to the cell walls were also found in the extracellular environment.

While bioinformatics tools are useful for predicting cellular function and localization, considerable variation exists in the number of proteins that were predicted to be extracellular, cytoplasmic, or membrane bound. This is expected because each of these bioinformatics tools uses different algorithms and assumptions in their predictions. Further empirical studies are therefore required to verify the precise location of proteins for which conflicting predictions are noted.

### Immunogenic extracellular proteins

The use of sera from human patients infected with cutaneous anthrax confirmed the high immunogenicity of PA in the secreted proteins of the toxigenic but non-encapsulated *B. anthracis *strain RA3R (pXO1^+^/pXO2^-^). Although several mass and charge variants of PA were detected, the most immunogenic are the 63 and 83 kDa charge variants. In addition to PA, the major component of AVA (anthrax vaccine adsorbed), enolase and transketolase were found to be highly immunogenic. AVA is an alum precipitate prepared from *B. anthracis *culture filtrates and is a licensed vaccine currently used to protect humans against anthrax. Enolase was also described previously as part of the AVA culture filtrate [29]. AVA preparations contain contaminating proteins whose benefits are not known, but the presence of the highly immunogenic enolase in this formulation might contribute to its protective immunity over a vaccine containing just PA. The membrane proteins Sap and EA1 were also found in the secretome by Chitlaru *et al*. [6] and Antelmann *et al*. [7] as the most abundant extracellular cell wall proteins. Both proteins were reported as major surface antigens and potential vaccine carriers *in vivo *[30,31]. EA1 and Sap are also major components of AVA [29] and are associated with a variety of functions ranging from evasion of host recognition, cell adhesion and resistance, and phagocytosis [1,32,33]. Interestingly no reactivity to these proteins was observed using sera from patients with subcutaneous anthrax infection. Overall fewer immunogenic proteins were detected using human sera from patients infected with cutaneous anthrax than in a recent study by Chitlaru *et al*. [30] who used experimentally challenged rabbit and guinea pig sera. There is a quantitative and qualitative difference between sera from recovering humans and sera from multiple challenged animals. Since with human sera enolase and transketolase were major immunogens beside PA, they might be promising candidates for next generation anthrax subunit vaccines besides the newly identified proteins using animal sera [30].

## Conclusion

The work presented here is the first description of the time course of protein secretion for *B. anthracis *grown under host-simulated conditions. The combined use of two types of mass spectroscopy led to the identification of 275 proteins and their secretion patterns during the exponential growth phase of *B. anthracis*. While the secretome contained several predictable proteins (*e.g*. PA, LF, Sap, and EA1) many proteins were identified which would not be expected in the secretome such as proteins involved in energy metabolism and protein translation. The discovery of proteins in the secretome that are traditionally thought to be strictly cytosolic has often been assumed to result from contamination. However, this may not be true since cytosolic proteins, such as aldolase, enolase, elongation factor G, and various dehydrogenases, have also been detected in the secretome of group A streptococci [34], mycobacteria [35,36], and *B. subtilis *[37]. Some proteins may be cytoplasmic at one point of the cell cycle and secreted via pathways which are yet to be understood during other stages of the cell cycle. While prediction software are a good tool to characterize a large group of proteins, different algorithms give diverging results. It is far more difficult to accurately predict a precise location within a cell of non-classical secretory proteins than to recognize proteins which are secreted by a signal peptide. The ultimate proof is to empirically validate their results. This study helps to bridge the gap between pure *in silico *prediction and *in vivo *observation.

This study also identified the major immunoreactive proteins of the *B. anthracis *secretome using 2-DE Western blot analysis from humans infected with the pathogen. These proteins included the expected PA as well as enolase and transkelolase. The immunoreactive secretome proteins contribute to the list of other *B. anthracis *immunogenic proteins that were idenfied in other subproteomes or life-stages of the infectious agent. This knowledge will ultimately lead to the development of a more-specific, safer, and highly efficacious vaccine against *B. anthracis*. In addition, the identification of early high abundance secretome proteins may aid in the development of detection and diagnostic kits for those cases, where the direct capture of the pathogen is not possible.

## Methods

### Bacterial strain and culture conditions

An attenuated derivative strain of *B. anthracis*, RA3R (pXO1^+^/pXO2^-^), was used. A loop was streaked on BHI agar overnight (16 hr) at 37°C. A single colony was transferred to 2 ml R medium [4] and approximately 1 ml of the resulting suspension was immediately transferred in 100 ml R medium supplemented with 0.25% [wt/vol] glucose. The flask was mixed gently and fitted with a BugStopper (Whatman Inc., Clifton, NJ) sterile venting closure. The culture was incubated at 37°C with shaking at 120 rpm for 5 to 6 hr. Following this growth period, 5 ml of the culture was transferred to a 250-ml sterile, vented, canted Falcon tissue culture flask containing 70 ml R medium with 0.25% [wt/vol] glucose and 0.85% [wt/vol] sodium bicarbonate. The composition of the R-medium in mg/l is: L-tryptophan, 35; glycine, 65; L-cystine, 25; L-tyrosine, 144; L-lysine, 230; L-valine, 173; L-leucine, 230; L-isoleucine, 170; L-threonine, 120; L-methionine, 73; L-aspartic acid, 184; sodium L-glutamate, 612; L-proline, 43; L-histidine-hydrochloride, 55; L-arginine-hydrochloride, 125; L-phenylalanine, 125; L-serine, 235; thiamine-hydrochloride, 1.0; CaCl_2 _2H_2_0, 7.4; MgSO_4 _H_2_0, 9.9; MnSO_4 _H_2_0, 0.9; K_2_HPO_4_, 3,000; uracil, 1.4; and adenine sulfate, 2.1, pH 8.0. The culture was grown for 6 to 16 hr at 37°C under 5% CO_2 _in a humid incubator according to Ristroph *et al*. [4] to simulate conditions encountered in the host. The pH at the end of the incubation period rises to pH 8.15. During this time the cells are in the exponential growth phase, between lag- and stationary- phase. Uninduced cultures were grown in R medium without the addition of sodium bicarbonate and without CO_2 _supplementation.

### Preparation of extracellular protein fraction

Culture filtrates (secretomes) were collected at 6 hr (early log phase), 10 hr (mid log phase), and 16 hr (late log phase) time points based on the growth curve for *B. anthracis *in R medium, as determined by OD_595 _readings. The culture filtrates were centrifuged for 10 min and the supernatant containing the extracellular proteins was passed through a 0.45-μm filter to remove any suspended vegetative cells. The culture filtrates were concentrated using Jumbosep, Macrosep, and Microsep filters with a 10 kDa cutoff (Pall Inc., East Hills, NY). Ice-cold trichloroacetic acid (TCA; Sigma Chemical Co., St. Louis, MO) was then added to a final concentration of 10% TCA (vol/vol), chilled on ice for 45 min, and then centrifuged for 45 min. The resultant pellet was washed with 3 ml acetone and centrifuged. The pellet containing the secretome proteins was resuspended in 100 μl of 7 M urea, 2 M thiourea, 1% 3-(4-heptyl)phenyl-3-hydroxypropyl-dimethylammoniopropanesulfonate, and 40 mM Tris prior to IEF. The protein concentration of the extracellular protein extract was determined using the Bio-Rad Protein Assay kit (Bio-Rad Laboratories, Hercules, CA). Bovine serum albumin was used as the standard for determination of protein concentration.

### 2-DE and Western blot analysis

2-DE was carried out with the ElectrophoretIQ3 system and following the manufacturer's protocols (Proteome Systems, Woburn, MA). One-hundred μg of proteins were separated by IEF on 11 cm (pH 4 to 7) linear IPG strips. After 12 hr rehydration, the following focusing parameters were applied: 50 μA per strip, linear voltage increase over 8 hr from 100 V to 10,000 V, and then held at 10,000 V for 10 hr. After IEF, IPG strips were equilibrated in equilibration buffer and applied onto a 6–15% gradient SDS-PAGE. Gels were electrophoresed for 1.5 hr at 500 V and stained with Sypro Ruby (Sigma-Aldrich, St. Louis, MO) for gel analysis or with ProteomIQ Blue (Proteome Systems) for MALDI-TOF MS analysis. Four replicate 2-DE gels of each sample were used for computer analysis using Phoretix 2D Expression software (Nonlinear Dynamics) and MALDI-TOF MS. Following automatic spot detection on quadruple gels using Phoretix 2D Expression's detection algorithm, gels were manually warped and their common spots were matched to generate average gels.

Immunoblotting was conducted according to Towbin *et al*. [38]. Proteins on 2-DE gels were transferred to PVDF membranes using Towbin buffer (0.025 M trisma base in 0.192 M glycine) with 20% methanol at 100 V for 30 min. After transfer, the PVDF membrane was washed twice for 5 min each in 0.01% Tween-20 in PBS (PBST) and blocked with 0.2% I-Block (Tropix, Bedford, MA) for 1 hr. To identify immunogenic proteins, the PVDF membranes were washed three times with PBST for 5 min and then probed with a 1:1000 dilution of sera pooled from patients who recovered from cutaneous anthrax. The PVDF membrane was washed three times with PBST and incubated with 1:5000 dilution of appropriate secondary antibody. Chemiluminescent signals were visualized using the Western Lightning reagents (Perkin-Elmer, Wellesley, MA). Corresponding sera from uninfected humans were used as controls (Cambrex, Charles City, IA). Three replicate blots were used for computer analysis using the Phoretix 2D Expression software to identify the immunogenic proteins.

### In-gel trypsin digestion and MALDI-TOF MS

Protein spots were excised, washed, and trypsin digested from 2-DE gels according to manufacturer's instructions using the Xcise robotic workstation (Shimadzu Biotech, Columbia, MD). Briefly, gel plugs were washed with 50 mM ammonium bicarbonate and 50% acetonitrile (ACN), dried, and treated with 1.6 μg/ml of trypsin in 50 mM ammonium bicarbonate at 37°C overnight. Tryptic peptides were applied to a MALDI-TOF MS plate in a solution of 10 mg/ml alpha-cyano-4-hydroxycinnamic acid in 0.1% trifluoroacetic acid and 50% ACN. MS spectra were obtained using an Axima-CFR plus (Shimadzu Biotech) in a positive ion reflectron mode and analyzed against the theoretical spectra of *B. anthracis *strain Ames, using the Mascot Daemon software package (Matrix Science, Boston, MA). The search parameters were: maximum of one missed cleavage by trypsin, fixed modification of oxidation, charged state of + 1, and mass tolerance of ± 0.5 Da.

### Protein identification by LC MS/MS

All LC MS/MS analyses were performed using an Agilent 1100 nanopump system coupled with a Vydac C18 reverse phase column (75 μM) (Agilent, Santa Clara, CA) and a QTRAP 2000 LC MS/MS system (Applied Biosystems, Foster City, CA) outfitted with a nanospray source and controlled with Analyst 1.4.1 software. Proteins were prepared for digestion using heat denaturation and a modified organic-aqueous digestion method as described before [14]. Each digested and desalted sample was re-suspended in 10 μl of Buffer A (95% water, 5% ACN, 0.1% formic acid). Each sample (7 μl) was loaded onto the LC MS/MS system and analyzed using an independent data acquisition method with the following parameters: single enhanced mass spectra (EMS, 500–1500 m/z) from which the three most intense peaks were subjected to an enhanced resolution, from which ions with a charge state of + 2 to + 4 were subjected to an enhanced product ion [EPI (MS/MS)] scan. Once the three most intense peaks were subjected to downstream analysis, they were ignored for a period of 60 sec. LC gradient was 5–60% Buffer B (95% ACN, 5% water, 0.1% formic acid) over 35 min. General parameter settings are as follows (curtain gas: 15.00, collision gas: High, ion spray: 2200 v, interface heater: on, declustering potential: 30, entrance potential: 10, collision energy: 10, rolling collision energy). All MS/MS data were searched against the theoretical spectra of *B. anthracis *Ames using the MASCOT software (Matrix Science). The search parameters used were: maximum of one missed cleavage by trypsin, fixed modification of oxidized methionine, charge state of + 2 and + 3, an MS tolerance of ± 1.2 Da and an MS/MS tolerance of ± 0.8 Da. Only protein identifications that met or exceeded the minimal MOWSE score of 22 were included in additional file 1.

## Abbreviations

2-DE: two-dimension gel electrophoresis

ACN: acetonitrile

EF: edema factor

IEF: isoelectric focusing

LC: liquid chromatography

LF: lethal factor

MALDI-TOF: matrix assisted laser desorption/ionization time of flight

MS: mass spectrometry

MS/MS: tandem mass spectrometry

PA: protective antigen

SLH S-layer homology

## Competing interests

The author(s) declare that they have no competing interests.

## Authors' contributions

AW carried out the secretome preparation, data analysis, conceived the study, and helped drafting the manuscript. CVM drafted the manuscript and participated in design of the study. JPC and TA performed the MALDI-TOF and LC MS/MS analysis. RC performed the 2D Phoretix comparative analysis. CD and JW performed the 2D gel electrophoresis and Western blotting. SPK and ASK participated in the design of the study, and VGD participated in the design of the study and helped to draft the manuscript. All authors read and approved the final manuscript.

## Supplementary Material

Additional file 1 Secretome proteins of *B. anthracis *strain RA3R (pXO1^+^/pXO2^-^) at 16 hr, as identified by MALDI-TOF and LC MS/MS Click here for file
